# Head fat is a novel method of measuring metabolic disorder in Chinese obese patients

**DOI:** 10.1186/1476-511X-13-113

**Published:** 2014-07-12

**Authors:** Xing-chun Wang, Huan Liu, Yue-ye Huang, Hang Sun, Le Bu, Shen Qu

**Affiliations:** 1 Department of Endocrinology and Metabolism, Shanghai Tenth People’s Hospital, Tongji University, Shanghai, China; 2 Department of Clinical Medicine, Nanjing Medical University, Nanjing, China

**Keywords:** Obesity, Head fat, Regional fat, Lipid profile, Hyperuricemia

## Abstract

**Background:**

Body adiposity, especially ectopic fat accumulation, has a range of metabolic and cardiovascular effects. The aim of this study was to investigate the association between head fat and metabolic values in Chinese obese patients.

**Methods:**

Data of this cross-sectional study from 66 obese patients were collected. Fat distribution was measured by dual-energy X-ray absorptiometry, and data of body weight, body mass index (BMI), neck circumference (NC), waist circumference (WC), hip circumference (HC), visceral index, basal metabolism (BM), glucose metabolism, lipid levels, uric acid (UA) had been collected.

**Results:**

1) Head fat was significantly associated with BMI, WC, HC, visceral index, BM, total fat and total fat excluding head fat in both males and females (*p* < 0.05). Head fat was positively correlated with upper limb fat, trunk fat, weight, fasting plasma C peptide, fasting plasma insulin and UA in women(*p* < 0.05), and the association was not statistically significant in male (*p* > 0.05). Head fat was positively corrected with NC in males (*p* < 0.05) but not females (*p* > 0.05). There was no significant correlation between head fat and fasting plasma glucose, total choleslerolemia, triglyceridemia, high-density lipoprotein cholesterol, low-density lipoprotein cholesterol and free fat acid in either gender (*p* > 0.05). 2) Receiver operating characteristic analysis showed that a head fat of 1925.6 g and a head fat of 1567.85 g were the best cut-off values to determine subjects with low high-density lipoprotein cholesterol and hyperuricemia respectively.

**Conclusions:**

Head fat accumulation was closely associated with increased body fat, hyperinsulinemia, hyperuricemia, and impared lipid profile, suggesting it might be used as an indicator for dyslipidemia and hyperuricemia.

## Background

Obesity is a public health problem that has become a global epidemic. According to the World Health Organization (WHO), there will be about 2.3 billion overweight people aged 15 years and above, and over 700 million obese people worldwide in 2015. Substantial literature has emerged to show that overweight and obesity are major causes of co-morbidities, including type II diabetes mellitus (T2DM), hyperlipidemia, hyperuricemia and cardiovascular disease. Body adiposity, especially ectopic fat accumulation, has a range of metabolic and cardiovascular effects.

Previous studies have already shown that overweight or obese patients who have the same amount of total body fat may have different risk factor profiles [1]. Therefore, regional fat distribution is better than the total fat in the prediction of cardiovascular disease and metabolic disease. As we know, the accumulation of abdominal fat also called central obesity is strongly associated with metabolic syndrome, T2DM, and cardiovascular disease [2-4]. Excess abdominal visceral adiposity is the key correlate of metabolic syndrome and upper body fat distribution is commonly associated with increased visceral fat and an abnormal metabolic profile [5-8]. In addition, previous data also suggest that fat accumulation peripherally such as in the lower extremities is inversely associated with adiposity-related biological factors and risk of metabolic syndrome [9]. The regional obesity also relates to obstructive sleep apnea syndrome (OSAS) as neck fat is one of risk factors of OSAS, and a predictor of the severity of OSAS [10]. Additionally, neck circumference (NC), which mainly reflects the fat deposit around respiratory tract or subcutaneous fat in the neck, is a proxy for upper-body fat and a reliable screening measure for the identification of patients with abnormal fat distribution. NC can be used as a marker to reflect central obesity and cardio-metabolic syndrome [11-13].

The relationship between regional fat accumulation of head fat and metabolic risks in obese patients remains unknown. Thus, we conducted this study to investigate whether head fat is related to regional fat distribution,metabolic values, and whether head fat can as used as a predictor for dyslipidemia, impaired fasting glucose, hyperinsulinemia and hyperuricemia in obese Chinese patients.

## Results

### Baseline characteristics of patients

The study sample consisted of 66 patients, 20 (30.3%) male and 44 (69.7%) female, with a mean age of 30.78 ± 9.66 years and a mean head fat of 1696.01 ± 336.10 g. The main characteristics of the study populations were shown in Table 1. There were significant differences in body weight, waist circumference (WC), visceral index, basic metabolism (BM) and total fat% between men and women (p < 0.01). Other anthropometry and metabolic variables were not significantly different between the genders (*p* > 0.05).

**Table 1:** Baseline study sample characteristics according to genders

**Parameters**	**Men**	**Women**	***p*-value**
**Number**	20	46	
**Age, years old**	29.45 ± 8.65	31.37 ± 10.10	0.463
**Head fat, g**	1829.43 ± 337.66	1638.01 ± 321.99	0.032
**Body weight, kg**	102.13 ± 19.74	83.37 ± 12.07	0.001
**BMI, kg/m**^**2**^	33.24 ± 4.46	31.85 ± 4.25	0.234
**NC, cm**	42.01 ± 4.66	38.30 ± 9.54	0.133
**WC, cm**	109.58 ± 14.69	99.50 ± 8.59	0.002
**HC, cm**	112.79 ± 11.13	107.48 ± 8.25	0.049
**Visceral index**	17.64 ± 6.37	12.19 ± 5.73	0.002
**BM, kcal**	2062.22 ± 339.91	1623.66 ± 189.49	<0.001
**FPG, mmol/l**	5.80 ± 1.75	5.43 ± 1.40	0.397
**Fasting insulin, uU/ml**	24.42 ± 13.78	23.75 ± 11.32	0.841
**Fasting CP, ng/ml**	3.85 ± 1.38	3.51 ± 1.26	0.358
**TCH, mmol/L**	4.90 ± 1.29	4.97 ± 0.96	0.824
**TG, mmol/L**	1.79 ± 0.78	1.75 ± 1.59	0.925
**HDL-C, mmol/L**	1.06 ± 0.26	1.24 ± 0.31	0.053
**LDL-C, mmol/L**	2.86 ± 1.22	2.84 ± 0.68	0.939
**FFA, mmol/L**	0.61 ± 0.22	0.61 ± 0.28	0.977
**UA, umol/L**	442.64 ± 92.08	368.43 ± 103.09	0.014
**Upper limbs fat, g**	5328.93 ± 1274.17	5493.33 ± 1304.78	0.637
**Lower limbs fat, g**	9843.77 ± 2838.54	10430.69 ± 2948.71	0.455
**Total limbs fat, g**	15172.70 ± 3746.38	15924.02 ± 3891.50	0.469
**Trunk fat, g**	17554.45 ± 3782.72	16420.15 ± 3596.75	0.251
**Total fat except head, g**	32731.45 ± 6787.01	32347.40 ± 6747.21	0.833
**Total fat, g**	34516.35 ± 6987.79	33989.90 ± 6875.77	0.758
**Total fat, %**	34.72 ± 4.41	41.30 ± 4.28	<0.001

BMI, body mass index; NC, neck circumference; WC, waist circumference; HC, hip circumference; BM, basic metabolism; FPG, fasting plasma glucose; TCH, total cholesterol; TG, triglyceride; HDL-C, high density lipoprotein-cholesterol; LDL-C, low density lipoprotein cholesterol; FFA, free fat acid; UA, uric acid.

Continuous data were described as means ± standard deviation.

*p-*values < 0.05 were considered significant.

### Correlation of head fat with anthropological and metabolic variables

Head fat was significantly associated with body mass index (BMI), WC, HC, visceral index, BM, total fat and total fat excluding head fat in both male and female (Table 2). Head fat was positively correlated with upper limbs fat, trunk fat, weight, fasting plasma C peptide, fasting plasma insulin in women, and the association was not statistically significant in male (Table 2). Head fat was positively corrected with NC in male (r = 0.667, *p* < 0.05) but not female. There was not significant correlation between head fat and fasting plasma glucose (FPG), total cholesterol (TCH), triglyceride (TG), high density lipoprotein-cholesterol (HDL-C), low density lipoprotein cholesterol (LDL-C) and free fat acid (FFA) in both genders (p > 0.05) (Table 2).

**Table 2:** Correlation of head fat with anthropological metabolic variables and fat distribution

	**Head fat**			
	**Men**		**Women**	
	**r**	**p-value**	**r**	**p-value**
**Body weight, kg**	0.540	0.014	0.607	<0.001
**BMI, kg/m**^**2**^	0.605	0.005	0.500	<0.001
**NC, cm**	0.667	0.003	−0.026	0.874
**WC, cm**	0.636	0.005	0.557	<0.001
**HC, cm**	0.637	0.004	0.415	0.008
**Visceral index**	0.699	0.001	0.568	<0.001
**BM, kcal**	0.599	0.007	0.623	<0.001
**FPG, mmol/l**	0.183	0.468	0.201	0.191
**Fasting insulin, uU/ml**	0.303	0.236	0.397	0.008
**Fasting CP, ng/ml**	0.496	0.051	0.396	0.010
**TCH, mmol/l**	−0.034	0.904	0.178	0.265
**TG, mmol/l**	0.264	0.341	0.153	0.225
**HDL-C, mmol/l**	−0.517	0.071	−0.235	0.138
**LDL-C, mmol/l**	0.346	0.298	0.200	0.211
**FFA, mmol/l**	−0.349	0.222	0.064	0.689
**UA, umol/L**	0.635	0.036	0.455	0.003
**Upper limbs fat, g**	0.502	0.024	0.533	<0.001
**Trunk fat, g**	0.546	0.013	0.420	0.004
**Lower limbs fat, g**	0.436	0.055	0.133	0.387
**Total limbs fat, g**	0.501	0.024	0.280	0.060
**Total fat except head, g**	0.581	0.007	0.386	0.008
**Total fat, g**	0.612	0.004	0.425	0.003

For list of abbreviations, see Table 1.

*p*-values < 0.05 were considered significant.

### Optimal cut-off points of head fat for abnormal lipid metabolism

The prevalence of high TG was 61.82%. Area under the receiver operating characteristic curve (ROC) was 0.641 for predicting high TG (*p* = 0.077). The prevalence of high LDL-C was 48.07% and area under the ROC curve was 0.493 for predicting high LDL-C (*p* = 0.941). The prevalence of high TCH was 32.14% and area under the ROC curve was 0.473 for predicting high TCH (*p* > 0.05). The prevalence of low HDL-C was 38.89%. 1925.6 g emerged as the optimal cut-off point of head fat for low HDL-C with a sensitivity of 57.1%, a specificity of 93.9% and the area under the ROC curve was 0.752 (*p* = 0.002, 95%CI: 0.613 ~ 0.890) (Table 3).

**Table 3:** Comparison of sensitivity, specificity, Youden index of high density lipoprotein cholesterol with different head fat point

**Head fat (g)**	**Sensitivity (%)**	**Specificity (%)**	**Youden index**
**1409.65**	95.2	18.2	0.134
**1577**	90.5	48.5	0.39
**1607.65**	85.7	51.5	0.372
**1644.55**	76.2	57.6	0338
**1668.25**	66.7	63.6	0.303
**1822.1**	61.9	84.8	0.467
**1925.6**	57.1	93.9	0.51

### Optimal cut-off points of head fat for IFG and hyperinsulinemia

The prevalence of impaired fasting glucose (IFG) was 22.95% in this obese population. Area under ROC curve is 0.553 (*p* = 0.548). The prevalence of hyperinsulinemia was 71.67% and area under ROC curve is 0.605 (*p* = 0.209).

### Optimal cut-off points of head fat for hyperuricemia

The prevalence of hyperuricemia was 67.74% in females. 1572.75 g was an optimal cut-off point of head fat for hyperuricemia with a sensitivity of 71.4%, a specificity of 80% and the area under the ROC curve was 0.738 (*p* = 0.035, 95%CI: 0.557 ~ 0.919) (Table 4). The prevalence of hyperuricemia was 38.89% in males and area under the ROC curve was 0.390 (*p* = 0.441).

**Table 4:** Comparison of sensitivity, specificity, Youden index of uric acid with different head fat point

**Head fat (g)**	**Sensitivity (%)**	**Specificity (%)**	**Youden index**
**1402.10**	90.5	20.0	0.105
**1528.00**	81.0	60.0	0.410
**1550.05**	76.2	70.0	0.462
**1572.75**	71.4	80.0	0.515
**1829.80**	38.1	100.0	0.381

## Discussion

Obesity is a public health problem that has raised concern worldwide. Accumulating evidence shows that regional fat distribution is a clinical indicator for the risk of T2DM and cardiovascular disease. Few articles focus on the association between head fat accumulation and metabolic disorders. In this study, we demonstrated that the regional accumulation of fat around the head was associated with classic obesity markers such as BMI, WC, HC, visceral index and BM in both genders. Head fat was also positively correlated with total fat, total fat excluding head fat, upper limb fat and trunk fat in females. Additionally, head fat correlated positively with fasting insulin, fasting C peptide and UA in female. We further revealed head fat might be a predictor for metabolic abnormalities including low HDL-C and hyperuricemia. Receiver operating characteristic analysis showed that a head fat of 1925.6 g was the best cut-off point to determine subjects with low HDL-C and a head fat of 1567.85 g was the best cut-off point to determine subjects with hyperuricemia in female. These results not only further confirmed the clinical significance of NC but also the metabolic impact of regional fat accumulation in the head.

Previous studies have shown that NC which represents neck fat deposit is a reliable anthropometric index to indicate central obesity [14]. In this study, we showed that head fat correlated positively with total fat and total fat excluding head fat in both genders. Additionally, head fat is closely related to upper limb fat and trunk fat in females. These results showed that head fat might also be associated with central obesity and upper body fat. The association between head fat and other regional fat was not significantly in males, probably because of the limited sample size.

The association between head fat and other regional fat indicated the importance of the head fat in predicting upper body fat, thus head fat might be used as a contributor to cardiometabolic risk. As we known, increased regional lipid content in the liver and muscle are associated with insulin resistance [15,16]. NC is not only associated with obesity and regional fat distribution, but also can be used for clinical screening of insulin resistance in high risk populations [17,18]. The analysis of our study revealed head fat was correlated positively with fasting insulin and fasting C peptide in females, suggesting head fat deposit might be a key feature of hyperinsulinemia and insulin resistance.

Elevated UA is a strong risk factor for cardiovascular disease and it plays an important role in the development of metabolic syndrome [19-21]. Studies indicate that weight gain is a risk factor for hyperuricemia while weight loss reduces the risk [22]. Hyperuricemia may be affected by differences in body fat distribution in obesity [23]. A previous study showed visceral fat may have a greater adverse effect on the metabolism of UA than BMI or subcutaneous fat [24]. Epicardial adipose tissue (EAT) thickness in children is associated with hyperuricaemia [25]. Similarly, our study also showed head fat was correlated with UA in female and 1572.75 gs is optimal cut-off point of head fat for hyperuricemia in females.

In addition, previous studies have shown that visceral fat mass is associated with elevated TG in obese women [26]. Visceral fat area has high positive correlations with TG level and a negative correlation with the HDL-C level in both genders [27]. Peripheral fat accumulation such as leg fat mass is favorably associated with cardiometablic risks factors [28,29]. NC what presents for peripheral fat of neck fat is associated with serum TCH, TG, increased LDL-C and decreased HDL-C in healthy adults [30]. Studies have also shown that NC is corrected with decreased HDL–C and increased TG [31-33]. Additionally, other studies reported that increased upper body subcutaneous fat is associated with increased LDL-C and decreased HDL-C [34]. The association between head fat and lipid levels in our study was not obvious. However, data analysis on lipid levels showed the prevalence of high TG, high LDL-C and high TCH were 61.82%, 48.07% and 32.14% respectively. The prevalence of low HDL-C was 38.89% and 1925.6 g emerged as optimal cut-off point of head fat for low HDL-C.

There were some limitations in this study. Firstly, the primary limitation of this study was the use of a cross-sectional design. Therefore, we cannot infer causality from our results. Secondly, the sample of the present study was relatively small. Finally, our result might not be applied to other racial or age groups because the studied subjects were predominantly Chinese adults. Therefore, further studies are necessary to prove these conclusions in larger sample size and with longitudinal studies.

## Conclusions

This study demonstrated that head fat was significantly associated with metabolic values among Chinese adults. Besides, receiver operating characteristic analysis showed the cut-off point of head fat to determine subjects with low HDL-C and hyperuricemia. Our findings might provide new insight into the role of head fat accumulation in the development of obesity-related diseases.

## Methods

### Sample population

A cross-sectional survey based on data from outpatient of Shanghai Tenth People’s Hospital was conducted among obese patients with BMI over 28 kg/m^2^ who had been seen in clinic between September 2011 and December 2013. The inclusion criteria included: 1) patients diagnosed with obesity (Obesity was defined as BMI ≥ 28 kg/m^2^) and 2) patients aged from 18–60 years old. People with medical illnesses such as clinical or laboratory evidence of cardiac, renal, liver or severe systemic diseases (e.g., cancer and heart failure) were excluded. A total of 229 obese subjects with measurement of dual-energy X-ray absorptiometry were enrolled for analysis. In addition to DEXA measurement, physical examination, including NC, WC, HC, and body weight, BMI, viscera index, BM, all subjects also underwent lab testing including fasting plasma glucose, fasting insulin, fasting C peptide, TCH, TG, HDL-C, LDL-C, FFA and UA. We excluded those patients lacking appropriate data (n = 163). After the appropriate exclusions, data of 66 patients were included in our analysis. The study protocol was approved by the ethics committee of Shanghai Tenth People's Hospital and written informed consent was obtained from each participant.

### Anthropometry

Weight, BMI, visceral index and BM were measured with light clothes and without shoes by Omron HBF-358 (Q40102010L01322F, Japan). WC was measured at the level midway between the lower rib margin and the iliac crest. NC was measured with head erect and eyes facing forward, horizontally at the upper margin of laryngeal prominence (Adam’s apple). HC was measured at the fullest point around the buttocks. These parameters were measured twice and the average was used for analysis.

### DEXA measurement

Body composition was measured using DEXA (Hologic QDR4500, USA). DEXA is a method for testing body composition and has high accuracy and good reproducibility, and is especially accurate for the determination of fat content and fat distribution [35]. DEXA was measured with the patient in the supine position. DEXA data including total fat%, upper limbs fat, lower limbs fat, trunk fat, total fat except the head, total fat, head fat. We calculated total fat in each limb.

### Lab testing

Blood samples were obtained after fasting over 8 hours for the measurement of lab testing including fasting plasma glucose, fasting insulin, fasting C peptide, TCH, TG, HDL-C, LDL-C, FFA and UA.

### Definition of dyslipidemia, impaired fasting glucose tolerance hyperinsulinemia and hyperuricemia

In this study, high TG was defined as fasting plasma TG ≥ 1.7 mmol/L, low HDL-C was defined as fasting HDL-C < 1.04 mmol/L,high LDL-C was defined as LDL-C ≥ 3.37 and high TCH was defined as TCH ≥ 5.18 mmol/L [36]. IFG was defined as fasting plasma glucose ≥ 5.6 mmol/l [37]. Hyperinsulinemia was defined as fasting plasma insulin ≥15 mU/L [38]. Hyperuricemia was defined as serum uric acid concentration ≥ 7 mg/dl (≥417 umol/L) in men, and ≥ 6 mg/dl (≥357 umol/L) in women [39].

### Statistical analysis

All data were analyzed using SPSS 17.0 software. All continuous data are presented as means ± standard deviation (SD). Quantitative data were analyzed by t-test between the two groups, the correlation between different variables were assessed using correlation analysis. ROC analyses was performed to assess the accuracy of head fat as diagnostic tests for dyslipidemia, IFG, hyperinsulinemia and hyperuricemia to determine optimal head fat cut-off in relation to dyslipidemia and hyperuricemia. Youden index (sensitivity + specificity −1) was used for determining the head fat cut-off point. All tests of significance were two tailed with a value of *p* < 0.05.

## Abbreviations

BMI: Body mass index; NC: Neck circumference; WC: Waist circumference; HC: Hip circumference; BM: Basal metabolism; UA: Uric acid; WHO: World Health Organization; T2DM: Type II diabetes mellitus; OSAS: Obstructive sleep apnea syndrome; DEXA: Dual-energy X-ray absorptiometry; TCH: Total cholesterol; TG: Triglyceride; HDL-C: High density lipoprotein cholesterol; LDL-C: Low density lipoprotein cholesterol; FFA: Free fatty acid; IFG: Impaired fasting glucose; SD: Means ± standard deviation; ROC: Receiver operating characteristic; FPG: Fasting plasma glucose; CP: C-peptide.

## Competing interests

The author’s declare that they have no competing interests.

## Authors’ contributions

XXW and HL conceived the study, analysed the data and wrote the manuscript. YYH and HS were involved in conducted the study. LB edited the manuscript. SQ organized the paper and approved the final version. All authors read and approved the final manuscript.
